# Water temperature variability at culvert replacement sites and river thermal impacts related to the removal of an old sediment pond: application on the Barnet Brook and a tributary of the Nerepis River (New Brunswick, Canada)

**DOI:** 10.1007/s10661-022-10117-5

**Published:** 2022-06-02

**Authors:** Daniel Caissie, Andy Smith

**Affiliations:** 1 Fisheries and Oceans Canada, Gulf Region, Science Branch, Freshwater Habitat Section, Moncton, NB Canada; 2 Environmental Services Branch, 5th Canadian Division Support Group, Department of National Defence, Gagetown Base, NB Canada

**Keywords:** Culverts, Stream temperature, Vegetation removal, Impact

## Abstract

Culverts are very important hydraulic structures for stream crossing, and they come in various shapes and materials. There are generally two different types of culverts, i.e., closed bottom and open bottom structures. In the present study, two closed bottom culverts have been replaced by open bottom structures (arch culverts) during the summer of 2018. The objective of the present study was to analyze water temperature variability along the impacted sites, one year after the replacement, i.e., 2019 to assess potential impacts of the streamside vegetation removal on the thermal conditions of these streams. Results showed a significant (*p* < 0.05) change in mean summer temperatures at both sites. Changes in stream temperatures at Barnet Brook were attributed to the removal of an old sediment pond, whereas changes in stream temperatures at the tributary of the Nerepis River were likely due to the removal of the streamside vegetation. Increases in water temperatures (> 4 °C) were more pronounced during low flow periods compared to high flow conditions at both sites.

## Introduction

Culverts are important hydraulic structures often used at stream crossing. As roads are being constructed for various purposes (e.g., forestry operations, construction of new highways, secondary roads, etc.), they often require the installation of culverts at stream crossings or within ditch systems. When using culverts as hydraulic structures, it is extremely important that they are properly designed; otherwise, these can be an impediment to fish passage and stream connectivity in general (e.g., Brenton et al., 2008; Briggs & Galarowicz, 2013; US Department of Agriculture, 2008). In fact, the connectivity of habitats is very important in maintaining healthy fish populations and proper ecosystem functioning in rivers (Erkinaro et al., 2017; Gibson et al., 2005). For example, migration is an essential part of the biological needs for many species, and if migration is delayed or disrupted, then fish and other organisms could potentially fail to reproduce in optimal habitats. Studies have shown that the type of culverts and the design can have an impact on fish movement, especially the ability of fish and other aquatic organisms to migrate upstream (e.g., Cenderelli et al., 2011). In order to properly migrate through culverts, fish need proper water depths, velocities (i.e., not too excessive) and proper flow attraction below the culvert (Goerig & Castro-Santos, 2017; Goerig et al., 2016). Trajkovic and Jenkins (2020) studied stream crossing within the Northwest Miramichi River in New Brunswick and found that most closed bottom culverts were either a potential barrier or a barrier to fish passage (based on the British Columbia culvert assessment protocol). In fact, when looking at 37 closed bottom culverts, 3% (1/37) were passable, 16% (6/37) were potential barriers, and 81% (30/37) were a barrier to fish passage. An additional 34 sites were on open bottom structures, and all open bottom structures were deemed passable to fish. Moreover, these open bottom structures were also located at high-habitat-value sites.

Studies have shown that culverts purely designed with hydraulic considerations tend to constrict the channel, increase velocities within the structure and generally have dissimilar conditions within the structure than those in natural channels (e.g., slope, substrate, depths, etc.; Schall et al., 2012). Some of the most noticeable disturbances are localized modifications of 1) stream slope, 2) loss of natural roughness or stream type substrate material within the culvert, and 3) an acceleration of local water velocities (generally within the structure, but also at the outlet). These disturbances often changes river morphology and impact fish passage. As a results of these issues, ecologically designed culverts are gaining popularity in North America and around the world (Cenderelli et al., 2011). These ecologically designed culverts have been adopted by US Department of Agriculture Forest Service, as a sustainable and long-term solution to maintain passage for all aquatic organisms at all life stages (US Department of Agriculture, 2008). These culverts, also known as stream simulation, are designed not only to pass fish but also other aquatic organisms, sediment, debris flow and woody debris, therefore mimicking the natural stream function, as much as possible.

Generally when close bottom culverts are replaced by open bottom structures, studies have shown an improvement in fish passage (Evans et al., 2015; Lawrence et al., 2014). However, very few studies have look at other factors related to culvert replacement, such as the potential impact related to water temperature variability in the vicinity of construction sites due to the removal of streamside vegetation (Cenderelli et al., 2011). Although the literature on this subject is scarce for culverts, the removal of streamside vegetation and its impact on watercourses has been well studied in forestry (e.g., Beschta et al., 1987; Gray & Edington, 1969; Lynch et al., 1984). In fact, changes in water temperatures and potential effect on aquatic habitat during timber harvesting have been well documented in a review by Beschta et al. (1987). In general, studies have shown that the removal of streamside vegetation tends to increase river temperature due to increased solar radiation reaching the stream (Brown & Krygier, 1970; Feller, 1981; Hewlett & Fortson, 1982; Holtby & Newcombe, 1982; Swift & Messer, 1971). For example, Hostetler (1991) observed an increase over 8 °C in a distance of less than 1.3 km of stream after the removal of streamside vegetation. Burton and Likens (1973) showed that successive opening of the streamside canopy contributed to increases in water temperatures in the range of 4 °C to 5 °C. However, they also pointed out that water temperatures tended to recover in the buffered sections of the stream. The partial removal of forest within the riparian buffer zone can also influence stream water temperatures, as reported by Feller (1981). These studies point to the fact the opening the stream canopy can increase the stream temperatures; however, such analyses needs to be carried out for other activities, such as culvert replacements.

The impacts of various activities on the riparian vegetation can impact the stream temperature which will ultimately impact on fish habitat and aquatic resources in general. Stream water temperature can influence a wide range of aquatic organisms from invertebrates (Cox & Rutherford, 2000; Hawkins et al., 1997) to salmonids (Lee & Rinne, 1980). For example, high stream water temperatures between 23 °C and 25 °C have been observed to affect the mortality of trout and their distribution within river systems (Bjornn & Reiser, 1991; Lee & Rinne, 1980). Juvenile Atlantic salmon can tolerate slightly higher temperatures than trout, in the range of 27 °C to 28 °C (Garside, 1973); however, behavioral changes are often observed at around 23 °C (Breau et al., 2011). As it relates to culverts and fish passage, water temperatures can also be important as it affects the swimming performance of fishes (Myrick & Cech, 2000).

Given the above observations, and the lack of knowledge on river temperatures related to culvert replacement, the objective of the present study is to quantify changes in stream temperatures (spatial and temporal variability) at two culvert replacement sites. Temperatures were not monitored before or during the culvert replacement; however, an upstream vs. downstream comparison analysis was carried out in order to study the spatial variability in stream temperatures. In fact, it is difficult at some of these sites to monitor conditions before construction activities as the whole channel is often modified in the impacted section. The two culverts are open bottom culverts located on tributaries of the Nerepis River, within the military base at Gagetown (New Brunswick).

## Materials and methods

### Study area

**Fig. 1:**
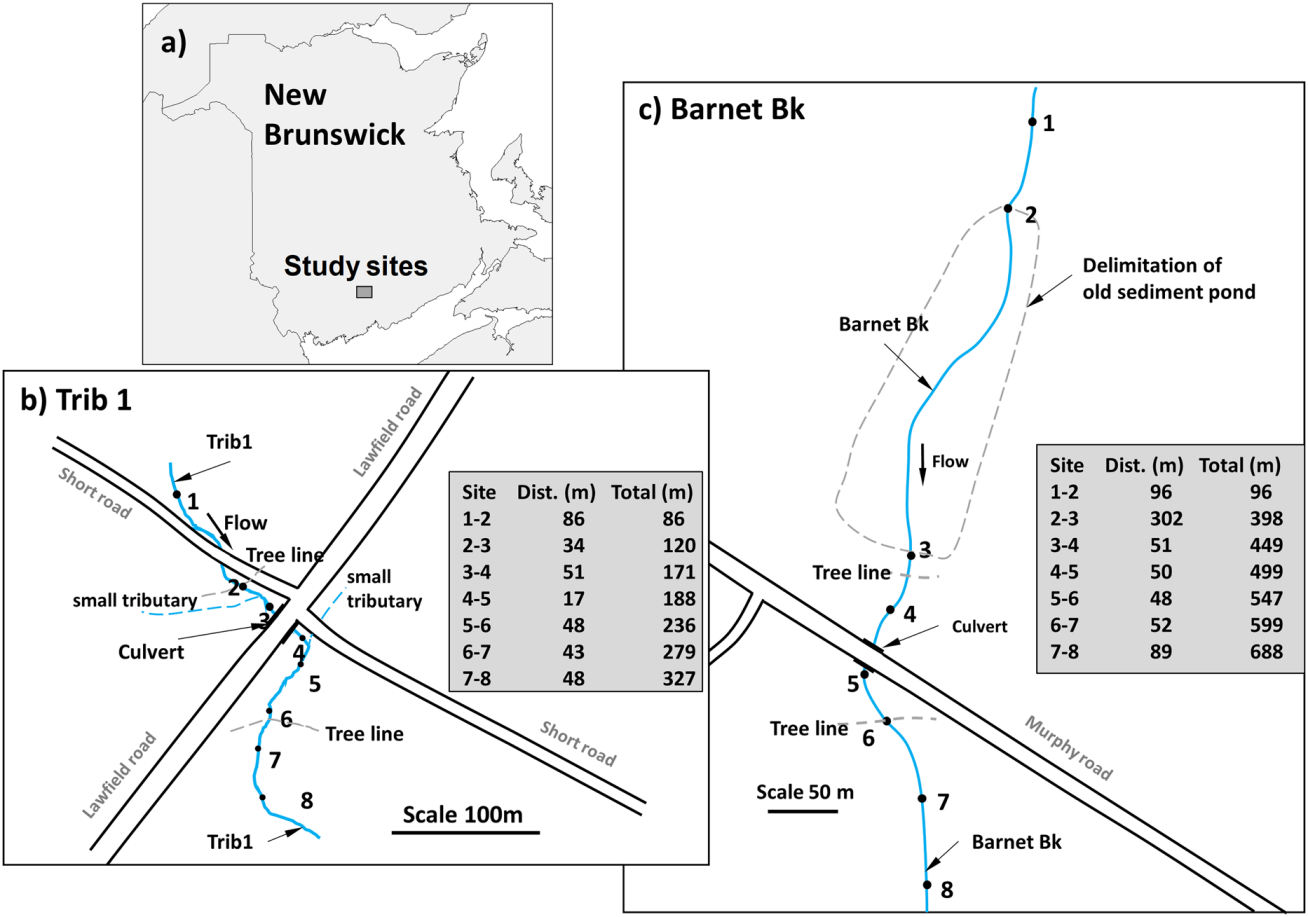
Location of culvert replacement sites in **a** New Brunswick (Canada), and **b** site map of the Nerepis River (Trib1), and **c** Barnet Brook

This study was carried out in two tributaries of the Nerepis River in 2019 where culverts were replaced in 2018 (one year after replacement). The first site is located on an unnamed tributary of the Nerepis River (thereafter named Trib1) at the intersection of Lawfield Road and Short Road (Fig. 1). This site is located at 45° 36′ 39″ N, 66° 16′ 46″ W. Trib1 has a drainage area of 0.84 km^2^ upstream of the road crossing and 1.11 km^2^ just below the road as a small tributary (0.27 km^2^) drains into Trib1 from a nearby ditch. Trib1 is typically between 1 and 1.5 m in width with a depth of 0.09 m. The second site is located on Barnet Brook (thereafter named BarnetBk) on Murphy Road (Fig. 1). This site is located at 45° 34′ 52″ N, 66° 14′ 53″ W. BarnetBk has a drainage area of 3.6 km^2^ above the stream crossing, and this watercourse is 2 m to 3 m in width with a depth of 0.15 m. Both sites are less than 4 km apart and therefore are subject to similar meteorological conditions (precipitation, air temperatures, etc.).

The culverts were replaced during the summer of 2018. Trib1 had a 1800 mm × 1800 mm concrete closed bottom arch culvert which was 30 m long. The culvert was replaced with an open bottom culvert (corrugated steel arch on concrete footings) with the following characteristics: arch of 6100 mm span × 3050 mm rise and was 22.3 m in length. The main fish species at this site are brook trout and slimy sculpin. Trib1 has been observed to run dry during dry summers. At the BarnetBk site, a corrugated steel pipe 2450 mm in diameter (12 m in length) was also replaced by an open bottom culvert. The open bottom culvert has the following characteristics: 9100 mm span, 4550 mm rise, 29.4 m long open bottom corrugated steel arch on concrete footings. The main fish species at this site are American eel, Atlantic salmon, blacknose dace, brook trout, creek chub, common shiner, finescale dace and white sucker. BarnetBk is a permanently flowing watercourse throughout the summer.

### Water temperature monitoring and water levels

At each site, 8 water temperature sensors were installed to monitoring the spatial and temporal variability in river temperatures. The sensors used were Vemco Minilog II with a manufacture stated accuracy of ± 0.1 °C for a temperature range of -5 °C to 35 °C with a battery life of 10 years. All 16 water temperature sensors were initially installed under shielded (shaded) conditions (i.e., under no direct solar radiation) for a period of 6 days (June 5 to June 10, 2019) to collect data in a common temperature environment for a cross-calibration analysis. The objective of the cross-calibration is to bring all sensors to the same mean temperatures before proceeding with the spatial analysis, see Caissie and El-Jabi (2019) for more details. Data were collected every 30 min for a total of 235 samples during the cross-calibration period. The mean water temperature of individual sensor during this period was between 12.95 °C and 13.02 °C which is reflective of the ± 0.1 °C stated manufacture accuracy (largest difference between two sensors was 0.07 °C). The mean overall water temperature (all sensors) was 13.00 °C during this 6-day period. Each sensor was thereafter adjusted by a systematic correction so that all temperature sensors would read the same mean temperature (13.00 °C) over the calibration period. After the cross-calibration, all sensors showed a typical root mean square error (RMSE) less than 0.007 °C, and the maximum difference in temperatures among sensors was less than 0.04 °C. This permits a high level precision comparison (< 0.04 °C) of stream temperatures among sites within each study reach (spatial differences) and among river systems (Trib1 vs. BarnetBk).

Following the cross-calibration of all 16 sensors, 8 sensors were installed at each of the study site (Trib1 and BarnetBk). Figure 1b shows the location of the water temperature sites at Trib1 and the distance between each site (sensors numbered from upstream to downstream). The total length of the studied stream reach at Trib1 is 327 m, and the riparian vegetation was removed (for the culvert replacement construction activities) between site 2 (tree line) and site 6 for a total distance of 150 m. Figure 1c shows the location of the 8 sensors at BarnetBk. At this site, an old sediment pond was present between sites 2 and 3; however, this pond was removed in 2015. The area outlined for the old sediment pond has very few trees and consisted mainly of tall grass and a braided river system, especially at the lower end of where the pond was. The section of BarnetBk where riparian vegetation was removed for construction-related activities is between sites 3–4 and site 6 which represents approximately 121 m. The total length of the studied reach at BarnetBk is 688 m between the first and the last water temperature sites. At site 4 of BarnetBk, a HOBO U20 water level recorder (0 to 4 m) was also installed for the duration of the study. This sensors has an accuracy of ± 0.075% FS (full scale) or ± 0.3 cm. As HOBO U20 sensors are sealed, a second unit, on the bank, next to site 4 was also installed for atmospheric barometric pressure correction. The water level recorder at BarnetBk was tied to a benchmark for reference, and stream discharges were measured at both Trib1 and BarnetBk during the summer period.

Data were also collected from a nearby hydrometric station operated by Environment and Climate Change Canada on the Nerepis River near Fowlers Corner (station 01AP006) for comparing water levels. This station is located at 45° 30′ 12″ N, 66° 19′ 08″ W and has a drainage area of 293 km^2^.

### Statistical analyses

To analyze changes in stream temperatures at different sites within each study reach, the Dunnett–Tukey–Kramer (DTK) pairwise multiple comparison tests were used (Lau, 2015). This pairwise multiple comparison test is used to analyze multi-level one-way experimental designs. It is designed to handle data where there are unequal sample sizes and population variance homogeneity cannot be assumed. In the present study, a significant level of 95% was used, and letters (A, B, C, etc.) were used to differentiate (graphically) sites that are significantly different. As such, any sites showing common letters are not significantly different, whereas others are.

## Results and discussion

### Daily water temperatures and water levels

**Fig. 2:**
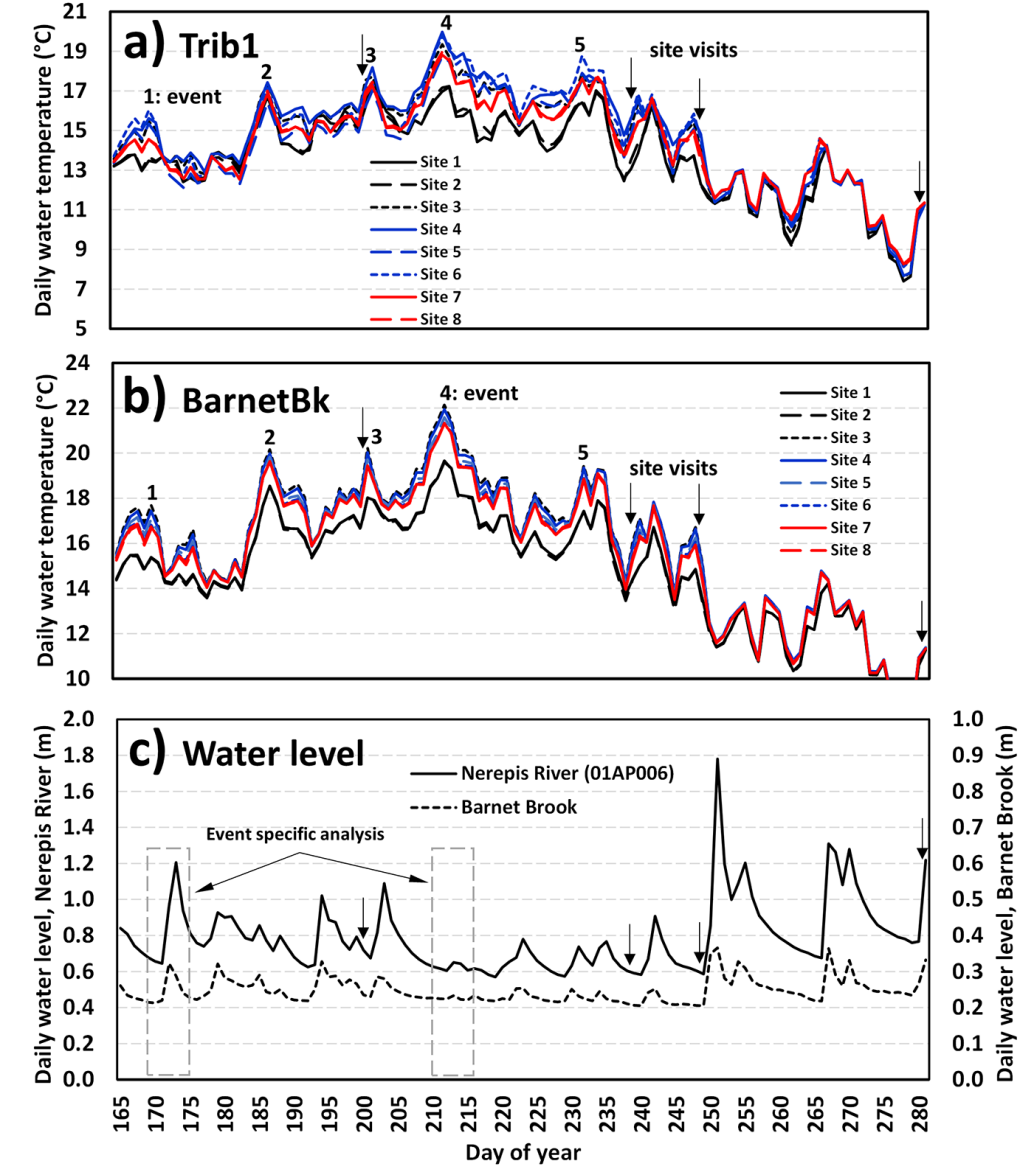
Mean daily water temperatures and water levels between June 14 (day 165) and October 8, 2019 (day 281). **a** water temperatures at Trib1 and **b** BarnetBk. **c** water levels at the Nerepis River (01AP006) and Barnet Brook. Arrows are indicating day of site visits and numbers are indicating specific high-temperature events

Figure 2 shows the daily water temperatures at Trib1 and BarnetBk (the two culvert replacement sites), as well as water levels (Nerepis River and BarnetBk) during the summer of 2019. Water temperatures showed similarities and differences among sites depending on flow conditions and the timing during the summer. Five high water temperature events are highlighted and discussed in more details below (identified by number 1 to 5; Fig. 2). The first high water temperature event occurred on day 170 (June 19) where water temperatures reached between 13.5 °C (site 1) and 16.0 °C (site 6) with a difference of 2.5 °C. During this event, the water levels at both the Nerepis River and BarnetBk were relatively low (0.656 m and 0.213 m, respectively). The second high water temperature event occurred on day 187 (July 6) and water temperatures varied between 16.4 °C (site 2) and 17.4 °C (site 4), with difference of 1 °C. Water levels were 0.715 m (Nerepis River) and 0.237 m (BarnetBk). The third high water temperature event occurred on day 202 (July 21) with temperatures between 17.0 °C (site 5) and 18.2 °C (site 4), a difference of 1.2 °C. The water levels during the third event were higher than the previous events at 0.816 m (Nerepis River) and 0.286 m (BarnetBk). The fourth high water temperature event occurred on day 212 (July 31), and water temperatures varied between 17.0 °C (site 1) and 20.0 °C (site 4) with a difference of 3.0 °C. This event represents the highest water temperatures of the summer and the largest spatial differences in river temperatures. The water levels during this event were 0.605 m (Nerepis River) and 0.224 m (BarnetBk) and occurring during the receding part of the hydrograph. Similar to the first event, sites 1 and 2 clearly showed lower temperatures compared to other sites. The last high water temperature event of the summer (event 5) was observed on day 232 (August 20), and temperatures varied between 16.4 °C (site 1) and 18.8 °C (site 6) with a difference of 2.4 °C. The water levels were 0.657 m (Nerepis River) and 0.223 m (BarnetBk). The mean reach temperature varied between 14.8 °C (day 170) and 18.7 °C (day 212). It can be observed from Fig. 2 that water temperatures at site 1 and site 2 of Trib1 were generally colder than the other sites during low water conditions. During higher discharges (high water levels), temperatures at all sites were very close to each other. This was most noticeable after the event on day 250 (September 7) and into the autumn period. Water temperature differences were more pronounced during low flow events with daily mean difference between 1 °C and 2.5 °C within reaches.

**Fig. 3:**
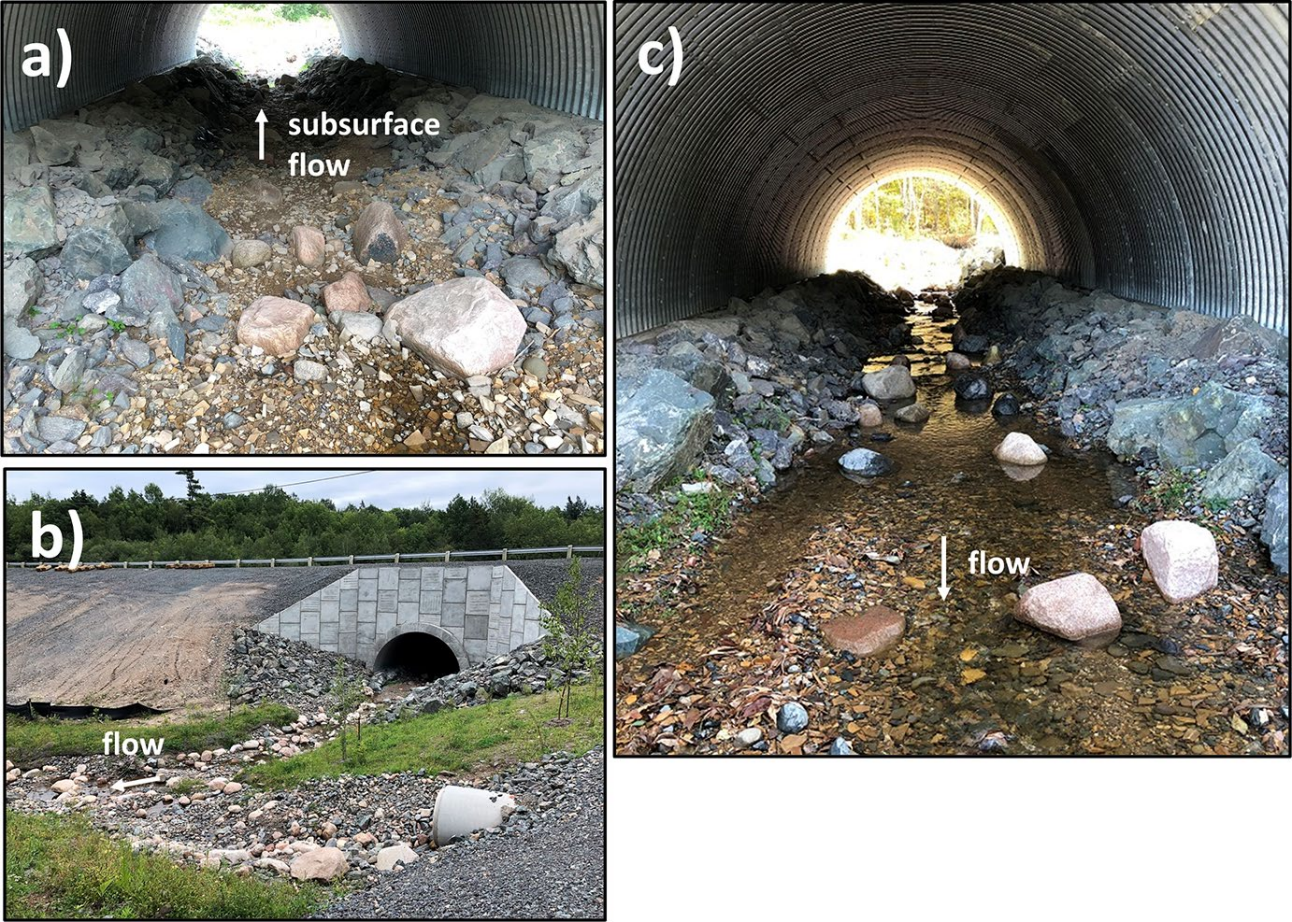
View of Trib1, **a** on August 26, 2019—dry section in the culvert, **b** on August 26, 2019—downstream of culvert, and **c** on October 9, 2019—inside the culvert to compare with panel (**a**)

Figure 2 also shows four site visits during the summer period (July 18, August 26, September 6 and October 8, 2019, indicated using arrows). The water levels during the site visits were 0.604 m (Nerepis River) and 0.211 m (BarnetBk) on August 26, 0.586 m (Nerepis River) and 0.205 m (BarnetBk) on September 6, and 1.218 m (Nerepis River) and 0.332 m (BarnetBk) on October 9. Trib1 showed discontinued flows (dry sections) on both August 26 and September 6. Figure 3a shows an example of such conditions on August 26 at Trib1 between site 3 and site 4 (inside the culvert), where subsurface flow conditions were observed. Figure 3b also shows the low water conditions downstream of the culvert on the same day. During these events, all water temperature sensors were under water, as sensors were installed in the deepest area of the stream, and Trib1 did not run dry throughout the reach. Notably, this site represented a combination of dry sections (subsurface flow) and flowing sections during low flows (Fig. 3a and b). Figure 3c shows Trib1 between site 3 and site 4 on October 9, where the reach became once again a continuous flowing reach.

**Fig. 4:**
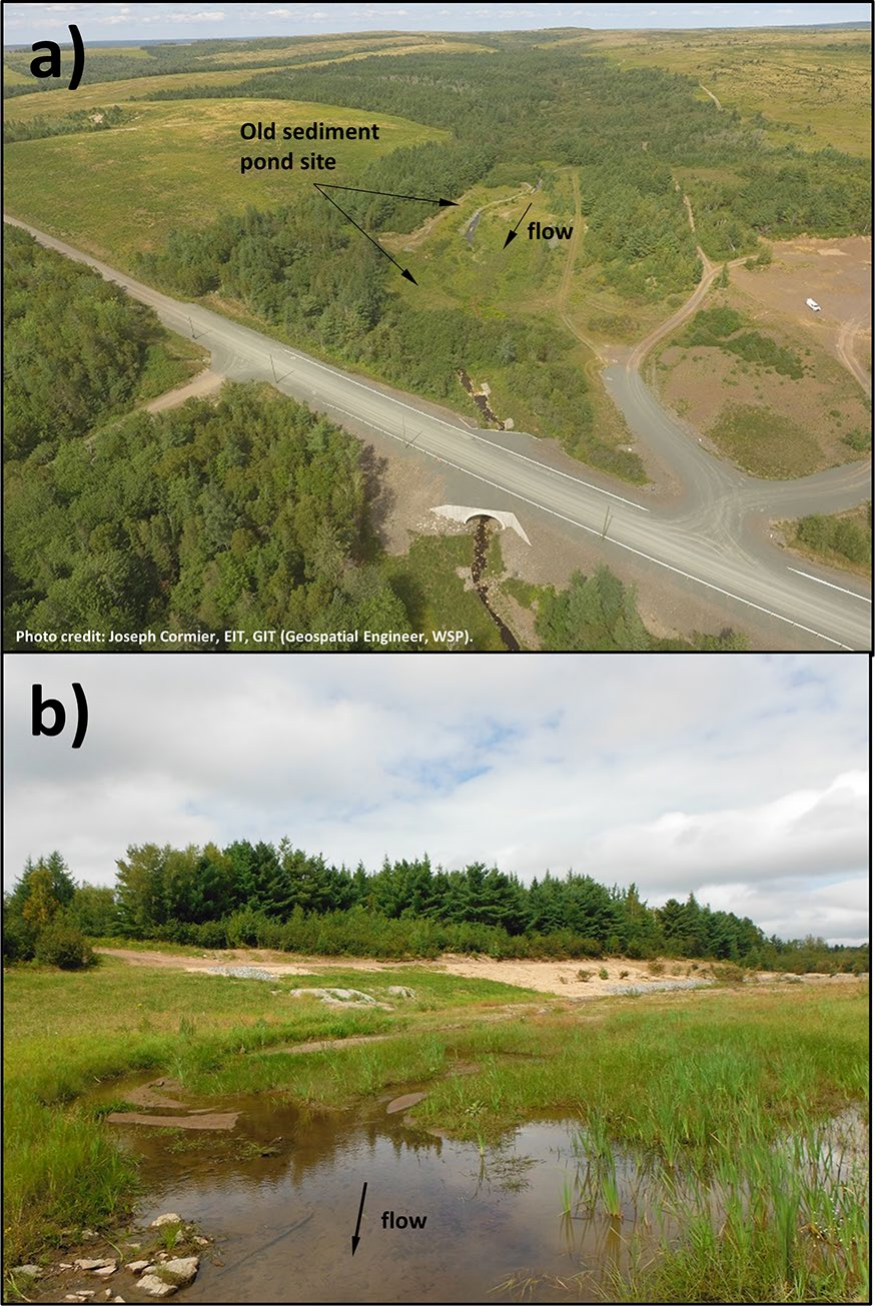
BarnetBk, **a** showing an aerial view of the site and the area of the old sediment pond, and **b** showing BarnetBk at the lower section of the old sediment pond, above site 3

The second study site was BarnetBk which experienced the same high-temperature events as in Trib1 (Fig. 2b). During the first high water temperature event (day 170; June 19), water temperatures were between 15.4 °C (site 1 and site 2) and 17.7 °C (site 3) with a difference of 2.3 °C. The second high water temperature event (day 187; July 6) showed water temperatures between 18.5 °C (site 2) and 20.2 °C (site 3), difference of 1.6 °C. During the third high-water-temperature event (day 202; July 21), water temperatures were between 17.8 °C (site 2) and 18.8 °C (site 3), a difference of 1.0 °C. The fourth high water temperature event occurred on day 212 (July 31), and water temperatures varied between 19.6 °C (site 1) and 22.1 °C (site 3) for a difference of 2.5 °C. This event also represented the highest water temperature of the summer. The last event occurred on day 232 (August 20), and temperatures varied between 17.4 °C (site 1 and site 2) and 19.4 °C (site 3 and site 4) with a difference of 2.0 °C. The mean reach water temperature varied between 16.6 °C (day 170) and 21.1 °C during the warmest event on day 212. The reach mean temperature was slightly higher at BarnetBk than Trib1, between 1.0 °C (day 187) to 2.4 °C (day 212, during the warmest event). Unlike Trib1 which showed times with similar temperatures among all sites (during higher flows), BarnetBk showed a consistent difference in temperatures between site 1 and site 2 compared to the other sites throughout the summer period. The late season water temperatures were similar among sites at both Trib1 and BarnetBk, i.e., after day 250 (September 7). At BarnetBk, site 1 and site 2 were above the influence of the old sediment pond reach, whereas all other sites were below, site 3 being just immediately below the influence of this reach. It is clear from these data that the old sediment pond area is influencing the thermal conditions within the study reach, and any local modifications of temperatures close to the culvert site would be confounded by this heating. As such, it will be difficult to tease out any water temperature variability due to the culvert replacement activities at BarnetBk. However, insights into the potential impact of the removal of this old sediment pond can be gained from the present study. Figure 4a shows BarnetBk where the old sediment pond was and the lower section of the studied reach at the tree line. This figure shows that this reach of BarnetBk is highly exposed to incoming solar radiation, which is most likely responsible for much of the heating observed between site 2 and site 3. Figure 4b shows the BarnetBk at the lower end (where the old sediment pond was), and where the river becomes slightly larger with slow moving waters. For instance, Mosley (1983) showed that braided rivers could be subject to very high water temperatures due to their shallow water depths and because they are highly exposed to meteorological conditions. These conditions are most likely experienced within this section of the BarnetBk.

Discharges were measured during some site visits at Trib1 and BarnetBk. The first discharge was measured at BarnetBk on July 18 (44.7 L/s), followed by August 26 at both BarnetBk (9.5 L/s) and Trib1 (1 L/s), and then on October 9 at BarnetBk (26 L/s). It is clear from these data that Trib1 showed very low discharges compared to BarnetBk. Notably, during all site visits BarnetBk showed flowing water throughout the study reach. On August 26, although a discharge of 1 L/s was measured at Trib1 below site 6, this watercourse showed discontinued flow conditions within the study reach (see Fig. 3, above).

### Mean summer water temperatures

**Fig. 5:**
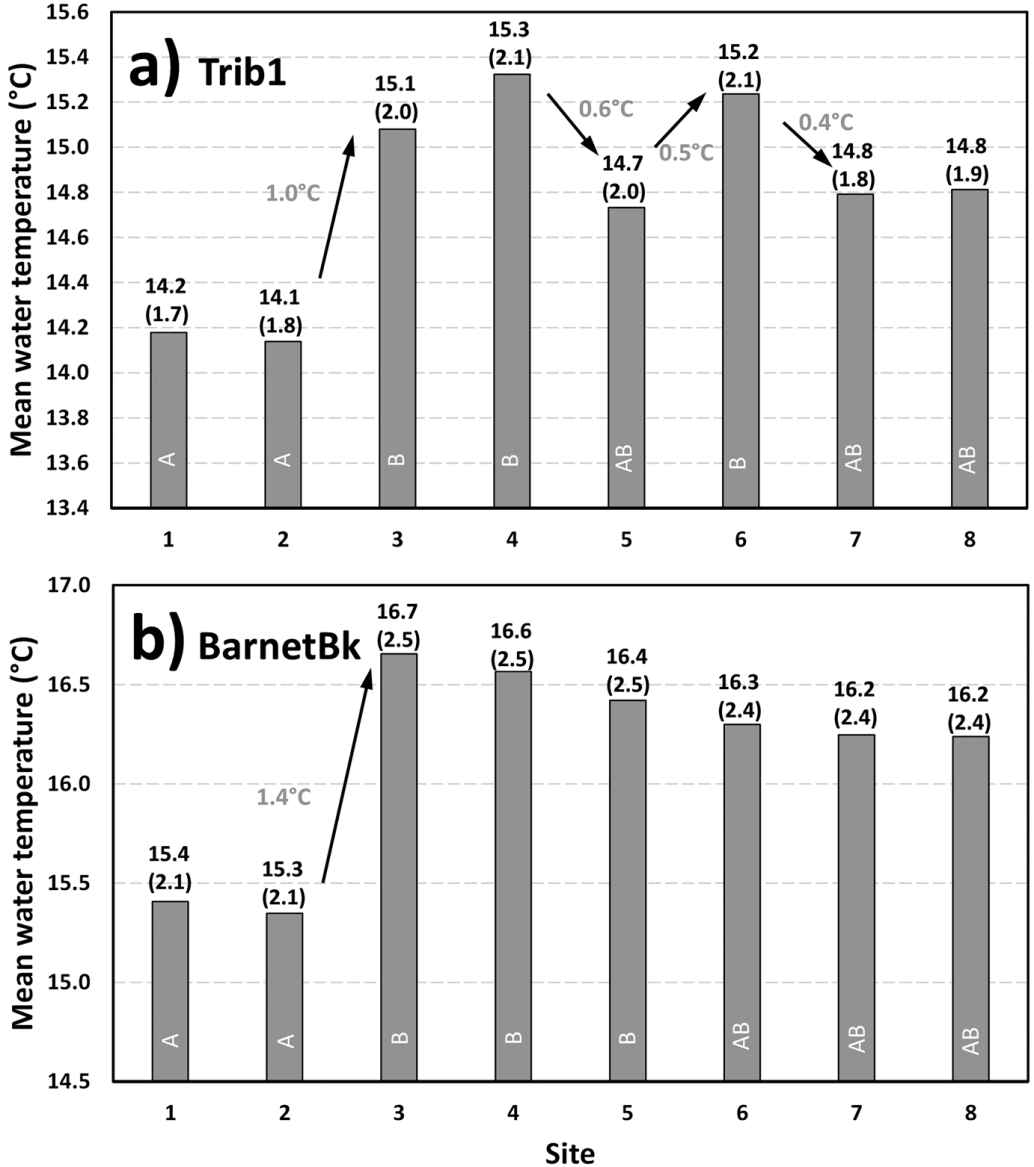
Mean temperature between June 14 and September 23, 2019, at all sites; **a** Trib1 and **b** BarnetBk. Values in parenthesis represent the standard deviation, and letters (A, B, etc.) represent the significant differences among sites (common letters are not statistically different; p < 0.05)

Following the analysis of daily water temperatures, the mean summer water temperatures were calculated at all sites for both Trib1 and BarnetBk, i.e., between June 14 and September 23, 2019. This period was selected to exclude autumn low water temperatures, i.e., when all sites showed similar temperatures which did not contribute much to the spatial variability analysis. Results of this analysis are shown in Fig. 5, where the spatial variability within each study site (at the reach level) can be observed. Results for Trib1 showed that site 1 and site 2 showed the coldest summer temperatures at 14.2 °C and 14.1 °C, respectively (Fig. 5a). The Dunnett–Tukey–Kramer (DTK) pairwise multiple comparison tests were carried out using daily water temperatures to study temperature differences among sites (as described in the method section). Letters (A, B, C, etc.) have been used in Fig. 5 to distinguish between significant and nonsignificant differences in temperatures (sites having common letters are not showing significant differences in temperatures whereas sites with different letters are). The DTK analysis showed six sites with significant differences in river temperatures at Trib1 (Fig. 5a; sites 3–1; 4–1; 6–1; 3–2; 4–2; 6–2). A significant increase in water temperature was observed between site 2 and site 3 (1.0 °C). Another increase was observed inside the culvert, i.e., between site 3 and site 4; however, this increase was not significant. The water temperature differences downstream of site 3 were not significant, as all site share the letter B (Fig. 5a). Also, sites 5, 7 and 8 did not show any significant differences in temperatures with sites 1 and 2. A small tributary between sites 4 and 5 was 2.3 °C colder than the Trib1 on July 18 during a site visit (using a handheld temperature sensor). The decrease in temperature between these sites (-0.6 °C), although not significant, was most likely due to this tributary. An increase in river temperature of 0.5 °C was observed between sites 5 and 6 (not statistically significant) followed by a decreases between site 6 (tree line) and site 7 (0.4 °C). Thereafter, the temperature essentially was the same between site 7 and site 8 (14.8 °C). Notably, Trib1 showed more variability within the construction reach where vegetation cover was removed and a significant increase in temperatures between sites 2 and site 6 (with the exception of site 5). Those sites that were within the undisturbed vegetation cover remained relatively stable, i.e., between site 1 and site 2, and between site 7 and site 8. Due to the small nature of this drainage basin, and the low volume of water to be heated (thermal capacity), it is clear that the solar radiation input in this open stretch of Trib1 influenced the local summer water temperatures between 0.4 °C and 1 °C (for summer means). This is consistent with the literature which shows that most of the heat gain in rivers is coming from incoming solar radiation (Brown, 1969; Hebert et al., 2011; Johnson, 2004).

Results for BarnetBk are shown in Fig. 5b. The most upstream sites (1 and 2) showed the coldest river temperatures during the summer period at 15.4 °C and 15.3 °C, respectively. The DTK results revealed six sites that showed significantly different river temperatures (i.e., sites 3–1; 4–1; 5–1; 3–2; 4–2; 5–2). There was a significant increase in river temperatures of 1.4 °C between site 2 (15.3 °C) and site 3 (16.7 °C). This reach (between site 2 and site 3, 302 m) experienced a significant increase in water temperatures most likely due to an increase in incoming solar radiation as a result of a lack of riparian vegetation shading the stream (Fig. 4). Downstream of site 3, the water temperature generally recovered slightly from the upstream heating but did not fully recovered in 290 m (distance between site 3 and site 8). The decrease in river temperatures between sites was between 0.1 °C and 0.2 °C but was not significant. Even when comparing water temperatures between site 3 and site 8 with a decrease of 0.5 °C, no significant differences were calculated. Given the influence within the river reach where the old sediment pond was, it is difficult to assessment any potential influences from removing the riparian vegetation locally at this culvert replacement site. However, the impact of removing the reservoir on this particular section of river can be assessed. For instance, removing the old sediment pond and creating an exposed section of river increased the temperature by 1.4 °C (for mean summer temperatures). It is generally accepted that artificial ponds (due to dams) in small watercourses typically increase water temperatures between the inflow and outflow. The issues increasing the temperature are likely due to increased exposure to solar radiation due to a lack of tall dense riparian forest, slowing of water velocity (increasing exposure time) and channel braiding (increasing area of water exposed to solar radiation). The unshaded section of the river and the slow moving waters, as a result of a wider section of this river (Fig. 4b), most likely contributed to the increase in river temperature (Mosley, 1983). The construction of a narrower and better defined channel, after the removal of the old sediment pond, could have potentially mitigated the increased heat within this section of the river. Changes during site-specific events are analyzed below.

### Event-specific water temperature variability

**Fig. 6:**
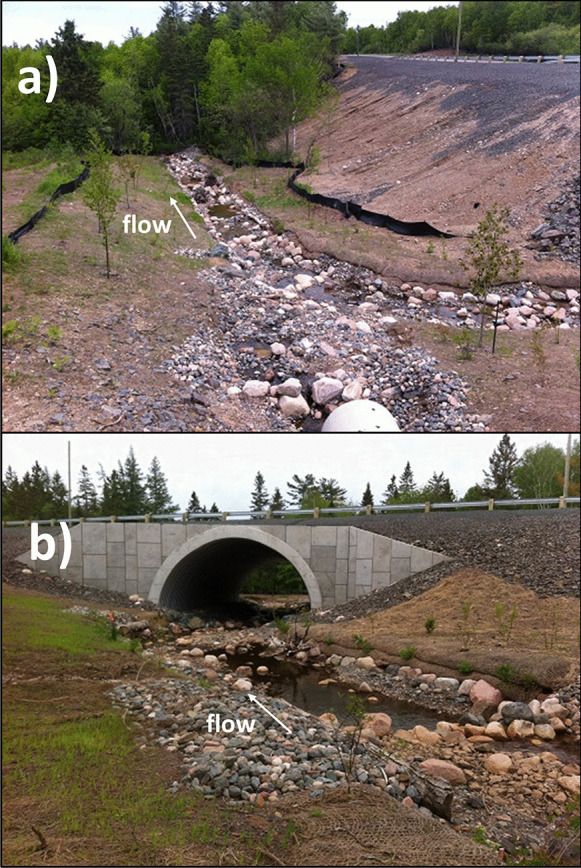
Study sites on June 13 (day 164) at **a** Trib1 downstream of the culvert and **b** BarnetBk upstream of the culvert

Important differences have been observed at different time scales, e.g., daily and summer mean water temperatures, as outlined above. This section looks at water temperature variability during specific events, as they can provide important information on both the spatial and temporal variability at finer temporal scales. Here, we will look at the diel variability as well as the maximum water temperatures on a daily basis rather than the mean over the summer to better assess potential heating/cooling between sites. Two events have been selected for this analysis, and they are identified in Fig. 2c (event-specific analysis). The first event occurred between day 169 (June 18) and day 173 (June 22). Permanent flows were observed at Trib1 during the site visit of June 13 (day 164, a few days before this event); however, flows were low within this study reach (Fig. 6a). Although water levels were low at BarnetBk, permanent flow conditions were present during this site visit (Fig. 6b). During this event, high flows were present after day 172 (June 21), as a result of 28 mm of precipitation in the region. The second event occurred between day 210 (July 29) and day 214 (August 2). This event represents the lowest flow conditions of the summer and the highest recorded water temperatures on day 212 (July 31). During the second event, air temperature reached 33 °C on July 30 (day 211) at the Gagetown DND meteorological station. During this event, Trib1 was most likely under discontinued flow conditions (dry sections), as later site visits showed dry sections of the stream under similar water levels.

**Fig. 7:**
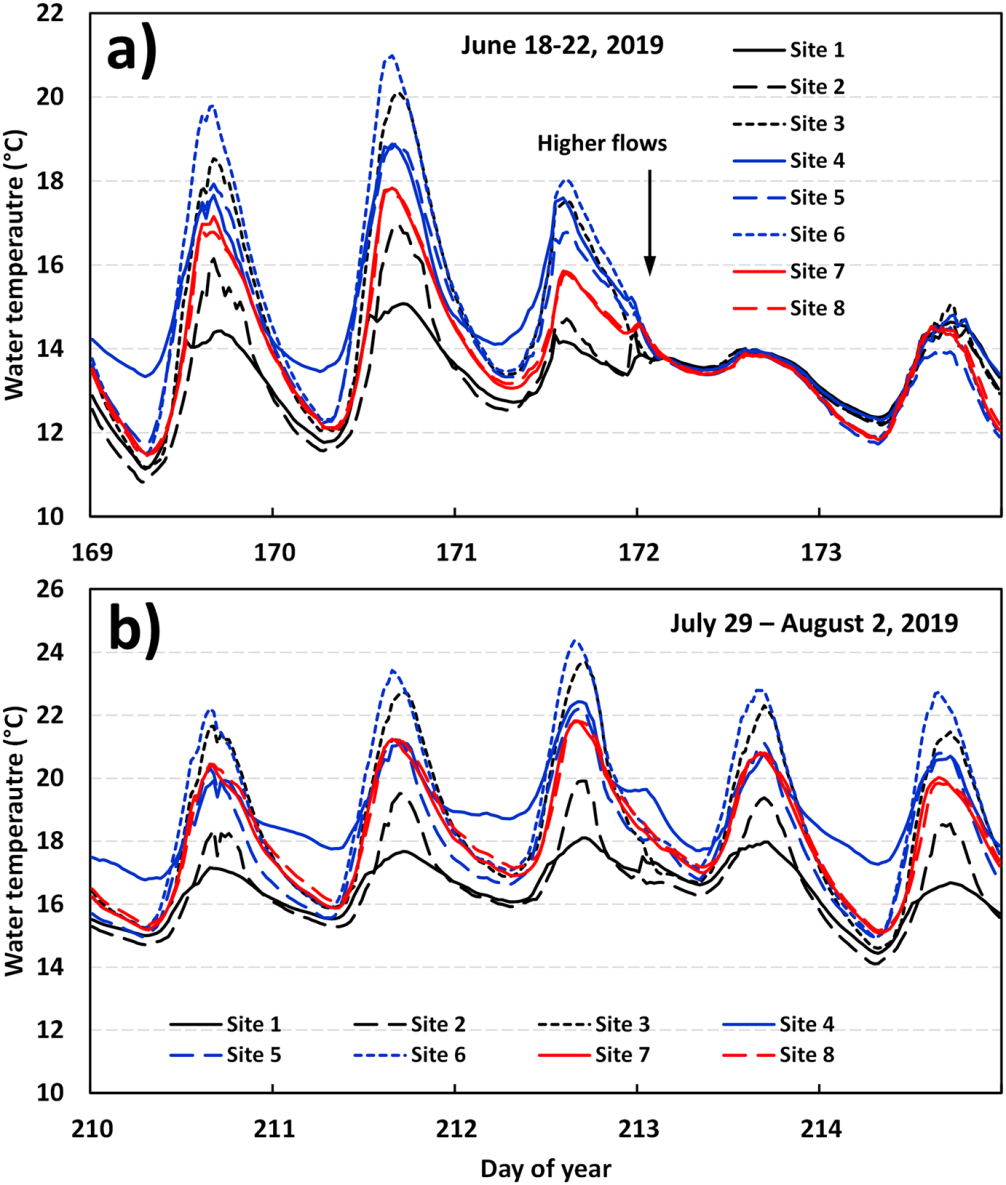
Water temperature variability at Trib1 during **a** June 18 and 22 (day 169–173) and **b** between July 29 and August 2, 2019 (day 210–214)

Results of the water temperature variability at Trib1 for the above specific events are presented in Fig. 7. In this figure, we will focus on maximum daily water temperatures, as minimum daily temperatures were similar among sites (with the exception of site 4). For instance, results show that site 1 is highly influenced by groundwater inputs with cold water temperatures that reached a maximum water temperature of only 14.4 °C (day 169). Other sites reached a maximum temperature of between 16.1 °C (site 2) and 19.8 °C (site 6) on this same day. Increase in water temperatures was noted between site 1 and site 2 for daily maximum temperatures (0.7 °C). However, a greater increase (local heating) was observed between site 2 and site 3 (between the tree line and the entrance of the culvert), maximum water temperatures of 16.1 °C and 18.5 °C (increase of 2.4 °C) on day 169, and 17.0 °C and 20.1 (increase of 3.1 °C) on day 170. A small recovery of water temperatures occurred within the culvert (between site 3 and site 4), and very little differences in temperatures were noted between site 4 and site 5. Another increase in water temperature was noted between site 5 and site 6 (the 48-m stretch of river along the road without vegetation). The increase in river temperatures between these two sites (at maximum temperatures) was 1.9 °C on day 169 and 2.1 °C on day 170. Hourly maximum temperatures generally occurred between 1400 and 1500 h during these days. An increase in discharge was observed during the morning of day 172 (June 21), and all water temperatures within Trib1 were relatively similar as shown in Fig. 7a.

The second analyzed event at Trib1 occurred between day 210 (July 29) and day 214 (August 2; Fig. 7b). This event includes the highest water temperatures of the summer (on day 212; July 31). Similar to the previous event, site 1 showed relatively cold water conditions and reaching maximum temperatures between 16.7 °C and 18.1 °C during this 5-day period. Site 2 showed slightly higher temperatures with maximum temperatures of between 18.4 °C and 19.5 °C (1.5–2 °C higher than site 1). Site 3 showed maximum daily temperatures between 21.5 °C and 23.7 °C (3–4 °C higher than site 2). As note previously, a slight recovery in water temperatures was noted inside the culvert, where water temperatures between site 3 and site 4 decrease by 1–1.5 °C. Stream temperatures between site 4 and site 5 were similar with differences less than 0.4 °C. An increase in maximum water temperatures was observed between site 5 and site 6. This increase was between 1.7 °C and 2.2 °C. Site 6 was the site where the highest river temperatures were recorded (24.4 °C on day 212). A recovery in maximum water temperatures was observed between site 6 and site 7 (decrease of approximately 2.2 °C). Water temperatures between site 7 and site 8 were almost identical (less than 0.1 °C). Interestingly, water temperatures between site 1 and site 6 increased by 6.3 °C (when comparing daily maximums) in 236 m during this high-temperature event. These results show how small tributary streams with a low discharge (low thermal capacity) can significantly increase during high summer temperature events. Burton and Likens (1973) showed similar increases in river temperatures (4–5 °C) when opening riparian buffer zone (between 50 and 75 m in length) in forest environments.

All sites showed a diel water temperature variability in the range of 2–7 °C; however, Trib1 showed two distinct patterns of diel water temperature variability. For instance, site 1 and site 4 showed the lowest diel variability (Fig. 7). Site 1 showed the lower diel variability (maximum—minimum = 2.1 °C) followed by site 4 (3.5 °C), and all other sites showed higher values (4–7 °C). The diel patterns at site 1 is indicative of a groundwater dominated streamflow, whereas the pattern at site 4 is indicative of a subsurface dominated flows but not necessarily groundwater, as minimum temperatures remain relatively high. Notably, the subsurface flow at site 4 showed a reduced diel variability; however during event 2 the mean water temperatures at site 1 and site 4 were 17.0 °C and 22.4 °C, respectively, on day 212. These results suggest that although site 4 was subsurface flow dominated, it was not groundwater dominated as an increase in mean temperature of 5.4 °C was observed between these two sites. Generally, when subsurface flow conditions are present with groundwater influences, then a cooling of temperatures occur as hyporheic flow upwells back into the river some distance downstream (Moore et al., 2005; Story et al., 2003). Conditions observed at site 4 (subsurface flow, but not groundwater dominated) could potentially be the results of the type of material used within the culvert, which may be deprived from fine sediments (< 2 mm). This could have resulted from a higher porosity thus favoring a subsurface flow, which is not necessarily connect to the groundwater flow system. At Trib1, it would be interesting to observe this site over the years to see if 1) the dried-up of sections remain the same at low flows and 2) to observed if the spatial water temperature variability changes with long-term exchange of fines within this section. For example, studies have shown that the bed material must be well graded and contain a sufficient quantity of fines (generally between 5 and 10%) so that the voids between larger rocks can be filled (Cenderelli et al., 2011). If the bed material does not include enough fines, flows can infiltrate into the bed (potentially creating subsurface flow conditions), unnaturally drying sections of the channel during critical low flow periods when aquatic species need to move or migrate. The role that fine material plays within reconstruction channels need to be further studied both from a short-term and long-term river sediment dynamics perspective.

**Fig. 8:**
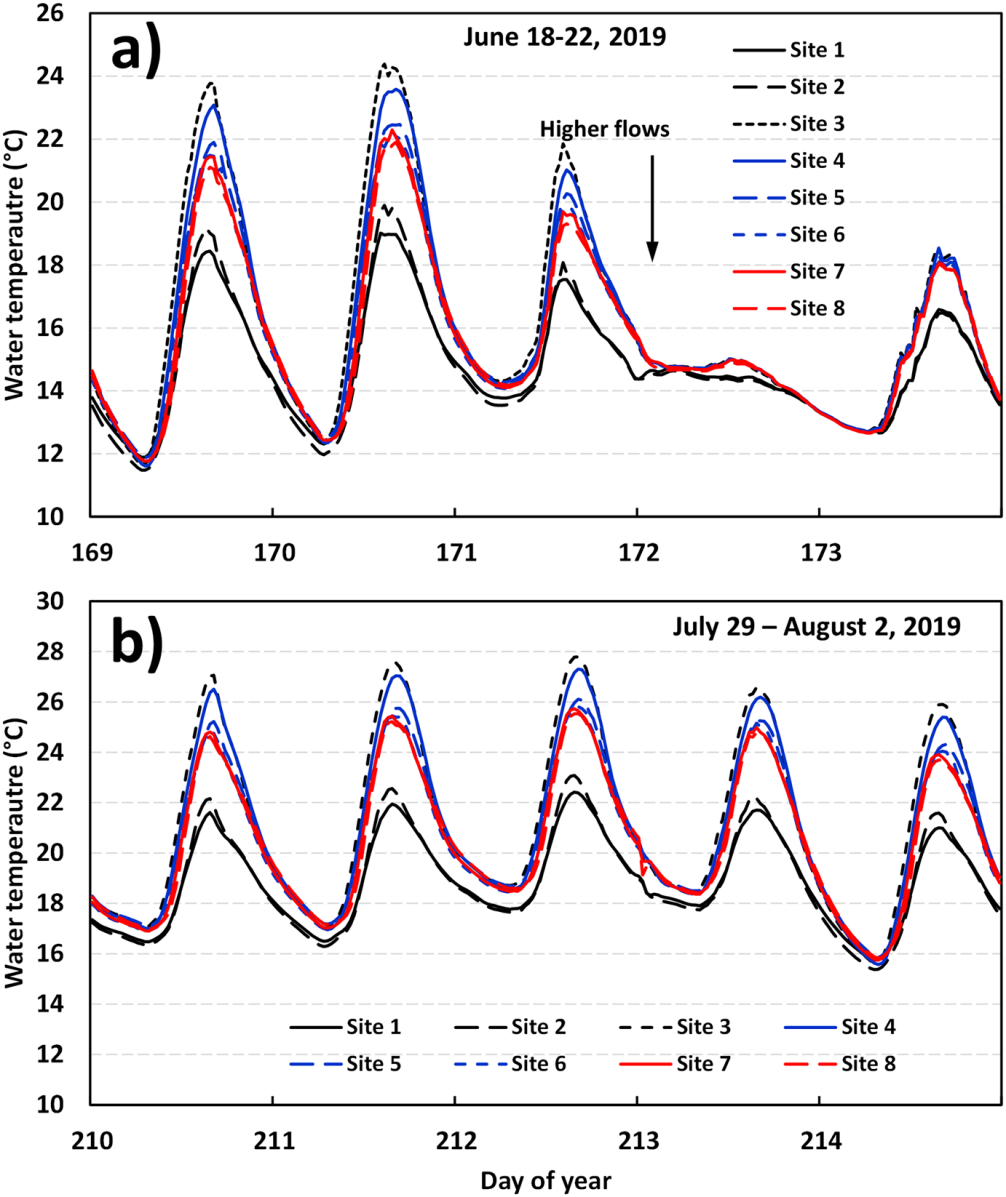
Water temperature variability at Barnet Brook during **a** June 18 and 22 (days 169–173) and **b** between July 29 and August 2, 2019 (days 210–214)

Figure 8 shows the results at BarnetBk for the same events (1 and 2) as those analyzed in Trib1. At this site, similar water temperatures were recorded between site 1 and site 2 (most upstream sites); however, the greatest differences in maximum river temperatures were observed between site 2 and site 3. In fact, maximum daily river temperatures at site 3 were 3.8 °C to 4.6 °C higher than site 2 (day 169–171; Fig. 8a) and reaching 21.8 °C to 24.4 °C at site 3. This increase in maximum daily water temperatures was most likely due to the highly exposed section of river to incoming solar radiation and the shallow river system between site 2 and site 3. Maximum water temperatures recovered between site 3 and site 6, but by only 1–1.2 °C. Maximum daily water temperatures between site 7 and site 8 were essentially the same. Similar to results observed at Trib1, BarnetBk showed very little water temperature variability among sites during the high discharges that occurred on day 172 and day 173. During the second event (Fig. 8b), a similar spatial pattern was observed, i.e., site 1 and site 2 being the colder sites followed by site 3, the warmest site. The increase in maximum daily water temperatures between site 2 and site 3 was in the range of 4.3 °C and 5.0 °C during this event. The highest increase was observed on day 211 (July 30) where water temperatures at site 3 reached 27.6 °C compared to 22.6 °C at site 2 (a difference of 5.0 °C). A slight recovery occurred between site 3 (27.6 °C) and site 8 (25.2 °C) on that day (recovery of 2.4 °C); however, water temperatures never reached values observed at site 1 and site 2. These results show that the significant increase of 5 °C in river temperatures between site 2 and site 3 (in 302 m) never recovered in the next 290 m (distance between site 3 and site 8). Although the heating and cooling reaches were of similar lengths, the above differences in temperatures, again, point to an input of incoming solar radiation between site 2 and site 3.

The results at both Trib1 and BarnetBk show that local water temperatures can reach lethal levels for aquatic resources and salmonids (> 25–28 °C) due to the opening of riparian vegetation either by construction related activities or when removing an old sediment pond. For instance, river temperatures upstream of the culvert replacement site at Trib1 were generally less than 20 °C, event during the warmest period of the summer, whereas temperatures reached 25 °C within the impacted zone. At BarnetBk, upstream temperature was slightly higher during the warmest period of the summer (~ 23 °C); nevertheless, stream temperatures reached 28 °C downstream of the old sediment pond. At BarnetBk, it was not possible to assess the impact of the culvert replacement activities due to the impact of the old sediment pond. This study showed the level of impact by exposing relatively small streams to solar radiation (by removing the streamside vegetation), even for relatively short distances (< 150 m at Trib1 and < 300 m at BarnetBk, old sediment pond section). A total recovery of river temperatures was also not reached at the downstream end of both study sites. In fact, stream temperatures at Trib1 remained 0.6 °C higher at the downstream site compared to the upstream site (Fig. 5). Similarly, stream temperatures at BarnetBk remained 0.8 °C higher at the downstream site compared to the upstream site.

## Conclusions

This study looked at water temperature variability at two culvert replacement sites within the Nerepis River (New Brunswick). The following points highlight the major finding of the present study.

1. Mean summer temperatures were impacted by the removal of streamside vegetation and flow patterns at the local scale within the Trib1 culvert replacement site.
2. Newly replaced culverts may potentially change the subsurface flow pattern locally, thus affecting the diel water temperature variability, as observed in Trib1.
3. The removal of an old sediment pond significantly increased average summer river temperatures in BarnetBk by 1.4 °C (over the study period, June 14 to September 23).
4. When looking at specific event, diel pattern and maximum daily temperatures, both study sites showed increase in stream temperatures between 4 °C (Trib1) and 5 °C (BarnetBk) with 150 m and 300 m, respectively.
5. The recovery in stream temperatures downstream of impact sites was slow and only recovered about 50% (~ 2.5 °C) of the increase due to solar radiation heating.

In conclusion, this study showed using upstream vs. downstream temperatures that both the replacement of a culvert and the removal of a sediment pond can impact the local water temperature variability. Observed increases in stream temperatures during the warmest period of the summer can exceed tolerable temperatures for salmonids within the study reach. This could potentially impact the level of stress encountered by fish, fish movement and even mortality among certain species.

## Data Availability

Data used during this study will be made available upon request to the corresponding author, Daniel Caissie (Daniel.Caissie@dfo-mpo.gc.ca).
